# Empagliflozin Ameliorates Free Fatty Acid Induced-Lipotoxicity in Renal Proximal Tubular Cells via the PPARγ/CD36 Pathway in Obese Mice

**DOI:** 10.3390/ijms222212408

**Published:** 2021-11-17

**Authors:** Chiang-Chi Huang, Chia-An Chou, Wei-Yu Chen, Jenq-Lin Yang, Wen-Chin Lee, Jin-Bor Chen, Chien-Te Lee, Lung-Chih Li

**Affiliations:** 1 Department of Internal Medicine, Division of Nephrology, Kaohsiung Chang Gung Memorial Hospital and Chang Gung University College of Medicine, Kaohsiung 833, Taiwan; herme381981@gmail.com (C.-C.H.); b9101088@cgmh.org.tw (C.-A.C.); leewenchin@gmail.com (W.-C.L.); chenjb1019@gmail.com (J.-B.C.); ctlee33@cgmh.org.tw (C.-T.L.); 2 Institute for Translational Research in Biomedicine, Kaohsiung Chang Gung Memorial Hospital and Chang Gung University College of Medicine, Kaohsiung 833, Taiwan; wychen624@gmail.com (W.-Y.C.); jyang@cgmh.org.tw (J.-L.Y.)

**Keywords:** CD36, empagliflozin, free fatty acid, PPAR-gamma, SGLT2 inhibitor

## Abstract

High serum levels of free fatty acids (FFAs) could contribute to obesity-induced nephropathy. CD36, a class B scavenger receptor, is a major receptor mediating FFA uptake in renal proximal tubular cells. Empagliflozin, a new anti-diabetic agent, is a specific inhibitor of sodium-glucose co-transporter 2 channels presented on renal proximal tubular cells and inhibits glucose reabsorption. In addition, empagliflozin has shown renoprotective effects. However, the mechanism through which empagliflozin regulates CD36 expression and attenuates FFA-induced lipotoxicity remains unclear. Herein, we aimed to elucidate the crosstalk between empagliflozin and CD36 in FFA-induced renal injury. C57BL/6 mice fed a high-fat diet (HFD) and palmitic acid-treated HK-2 renal tubular cells were used for in vivo and in vitro assessments. Empagliflozin attenuated HFD-induced body weight gain, insulin resistance, and inflammation in mice. In HFD-fed mice, CD36 was upregulated in the tubular area of the kidney, whereas empagliflozin attenuated CD36 expression. Furthermore, empagliflozin downregulated the expression of peroxisome proliferator-activated receptor (PPAR)-γ. Treatment with a PPARγ inhibitor (GW9662) did not further decrease PPARγ expression, whereas a PPARγ antagonist reversed this effect; this suggested that empagliflozin may, at least partly, decrease CD36 by modulating PPARγ. In conclusion, empagliflozin can ameliorate FFA-induced renal tubular injury via the PPARγ/CD36 pathway.

## 1. Introduction

Obesity is associated with several pathological conditions, including dyslipidemia, insulin resistance, diabetes mellitus (DM), and low-grade systemic inflammation [1]. An increased plasma free fatty acid (FFA) level, a common feature detected in patients with obesity or DM, has been implicated in the development of obesity-induced nephropathy [2]. Elevated FFA is predominantly observed in patients with obesity [3] and type 2 DM [4], as well as in animals fed a high-fat diet (HFD). Lipid accumulation in tubular epithelial cells, especially FFA, has been proposed to induce cellular lipotoxicity [5].

CD36, which belongs to the class B scavenger receptor family, is a single-chain transmembrane surface glycoprotein [6,7,8]. CD36, originally known as an FFA transporter, reportedly plays a role in fatty acid oxidation [9] and is expressed in numerous organs and tissues. For example, CD36 is expressed in various cell types within the kidney, including proximal tubular epithelial cells, podocytes, and mesangial cells [6]. In proximal tubular cells, advanced oxidation protein products, such as albumin and palmitate, increase CD36 expression [10]. Palmitate (C16:0), a saturated fatty acid, is one of the most abundant circulating FFAs [11]. Notably, CD36 has been associated with renal injury and inflammation in obesity-related metabolic syndromes [12,13,14]. Moreover, in a human study, the expression of renal CD36 was reportedly increased in the kidney tissue of patients with DM [5]. CD36 has also been shown to contribute to the progression of human diabetic nephropathy via palmitate-induced proximal tubular apoptosis and interstitial fibrosis [15]. Mice with endothelial cell-specific CD36 deficiency exhibited improved glucose tolerance and insulin sensitivity, and increased glucose uptake during fasting in heart, skeletal muscle and white adipose tissue [16]. In addition, mice with hepatocyte-specific deletion of CD36 also attenuates fatty liver and improved insulin sensitivity in HFD-fed mice [17].

Sodium-glucose co-transporter 2 (SGLT2) inhibitors are unique anti-diabetic drugs that promote urinary glucose excretion by increasing the glucose reabsorption threshold in the proximal tubule. In addition to glycemic control, SGLT2 inhibitors were found to confer renoprotective benefits in patients with type 2 DM, as documented in large clinical randomized trials (Empagliflozin, Cardiovascular Outcome Event Trial in patients with Type 2 Diabetes Mellitus [EMPA-REG OUTCOME] and CANagliflozin cardioVascular Assessment Study [CANVAS]) [18,19]. Furthermore, in the Dapagliflozin and Prevention of Adverse Outcomes in Chronic Kidney Disease (DAPA-CKD) trial, SGLT2 inhibitors were shown to offer renal protection not only in patients with DM but also in non-DM patients with proteinuria, suggesting that SGLT2 inhibitors confer renal protection beyond sugar-lowering effects [20]. SGLT2 is primarily located on the apical membrane of the proximal tubule, and SGLT2 protein inhibition could decrease renal lipid accumulation [21]. However, the underlying mechanism through which SGLT2 inhibitors regulate CD36 remains unclear, especially in the proximal tubular epithelial cells.

Previous studies have demonstrated that CD36 plays a crucial role in provoking an inflammatory cascade, including inflammasome activation in the renal proximal tubule [13,20]. As both SGLT2 and CD36 exist in the renal proximal tubules, the crosstalk between these two receptors during metabolic disorders is intriguing. Accordingly, we hypothesized that SGLT2 inhibitors might reduce the renal uptake of FFA by downregulating CD36 in an HFD mouse model. This study could help elucidate how SGLT2 inhibitors offer renal protection by modulating CD36 expression.

## 2. Results

### 2.1. Empagliflozin Attenuated HFD-Induced Body Weight Gain and Insulin Resistance

**Figure 1:**
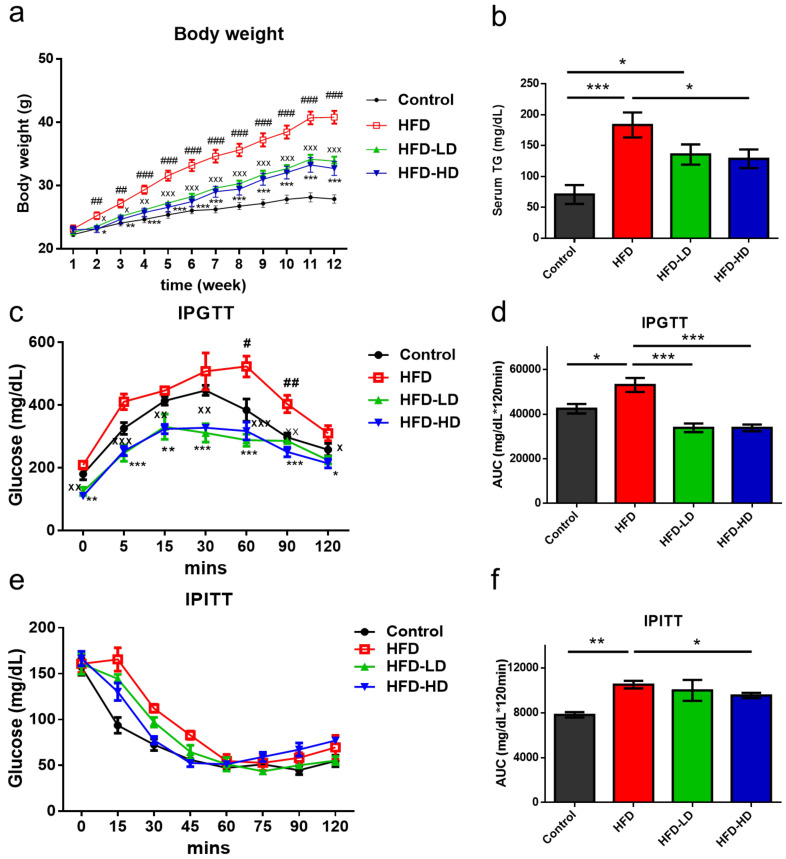
Empagliflozin attenuates metabolic parameters in HFD mice. (*n* = 6). Six-week-old C57BL/6 male mice were fed a control diet (CD, 10% from fat), HFD (58% kcal from fat), HFD+ low-dose empagliflozin (3mg/kg BW), or HFD + high-dose empagliflozin (10mg/kg BW) for 12 weeks. (**a**) Empagliflozin significantly attenuates HFD-induced body weight gain at the end of the 12-week period, regardless of empagliflozin dosage. (**b**) High-dose empagliflozin significantly decrease serum triglyceride level. (**c**,**d**) Both high and low dose of empagliflozin improves glucose tolerance but only high dose of empagliflozin significantly improved insulin tolerance (**e**,**f**). The histograms represent mean ± standard error (SE). Statistical analysis was performed with one-way ANOVA. ^#^ *p* < 0.05, ^##^ *p* < 0.01, ^###^ *p* < 0.001, HFD vs. control; ^X^ *p* < 0.05, ^XX^ *p* < 0.01, ^XXX^ *p* < 0.001, HFD-LD vs. HFD; * *p* < 0.05, ** *p* < 0.01, *** *p* < 0.001 (HFD-HD vs. HFD in (**a**,**c**), other meaning the significance between groups). AUC, area under the curve; HFD, high-fat diet; LD, low-dose empagliflozin; HD, high-dose empagliflozin; IPGTT, intraperitoneal glucose tolerance test; IPITT, intraperitoneal insulin tolerance test; TG, triglyceride.

Six-week-old C57BL/6 male mice were assigned to the following groups: (1) control diet (CD), (2) HFD, (3) HFD+ low-dose empagliflozin (3 mg/kg/day), and (4) HFD + high-dose empagliflozin (10 mg/kg/day). The mice were treated with empagliflozin or vehicle by single daily oral gavage for 12 weeks. As shown in Figure 1, HFD progressively increased the body weight (BW) of mice after three months of treatment. However, both high- and low-dose empagliflozin significantly attenuated HFD-induced BW gain at the end of the three-month period. High-dose empagliflozin seemed to exert similar effects to those mediated by low-dose treatment (Figure 1a). Serum triglyceride (TG) increased in HFD and high dose empagliflozin decreased TG level (Figure 1b). We next examined the metabolic effects of empagliflozin treatment on HFD mice; accordingly, intraperitoneal glucose tolerance test (IPGTT) and intraperitoneal insulin tolerance test (IPITT) were performed. HFD mice exhibited poor glucose tolerance, while both low-dose and high-dose empagliflozin treatment improved this parameter (Figure 1c,d). High-dose empagliflozin showed a superior improvement in IPITT than the low-dose treatment (Figure 1e,f). These findings suggested that empagliflozin could ameliorate HFD-induced insulin resistance.

### 2.2. Empagliflozin Attenuated HFD-Induced Lipid Accumulation in Mouse Kidneys

**Figure 2:**
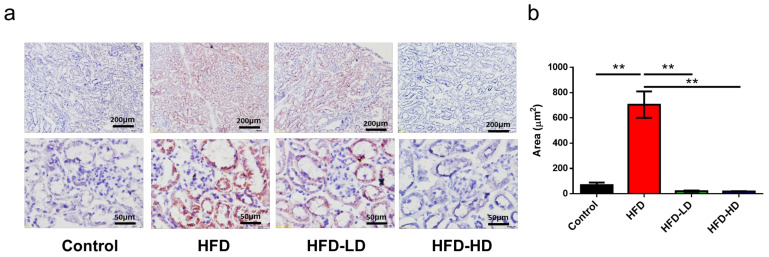
Empagliflozin attenuates lipid accumulation in the kidney of HFD mice. Six-week-old C57BL/6 male mice were assigned to the following groups: (1) CD, (2) HFD, (3) HFD + low-dose empagliflozin, or (4) HFD + high-dose empagliflozin for 12 weeks (*n* = 6 in each group). (**a**) In Oil red O-stained images, HFD significantly increases lipid accumulation when compared with CD, while empagliflozin attenuates lipid deposition in the kidney of HFD mice in a dose-dependent manner. Scale bars at upper panel = 200 μm, lower panel = 50 μm. (**b**) The histograms represent mean ± standard error (SE). Statistical analysis was performed with one-way ANOVA. ** *p* < 0.01 vs. HFD in each group. HFD, high-fat diet; CD, control diet; LD, low-dose empagliflozin; HD, high-dose empagliflozin.

Next, kidney sections were stained with Oil red O staining to evaluate lipid accumulation in the kidney. As shown in Figure 2, empagliflozin attenuated lipid uptake in the kidney, mainly in the tubular and interstitial areas, of HFD mice. Notably, high-dose empagliflozin was more potent than low-dose empagliflozin in preventing lipid deposition (Figure 2). Wang et al. also demonstrated that dapagliflozin, another SGLT2 inhibitor, reduced kidney triglyceride content in western diet-fed mice [22], suggesting that SGLT2 inhibitor can ameliorate HFD-induced fatty acid accumulation in the kidney.

### 2.3. Empagliflozin Attenuated HFD-Induced CD36 Expression and Cell Apoptosis in Mouse Kidneys

**Figure 3:**
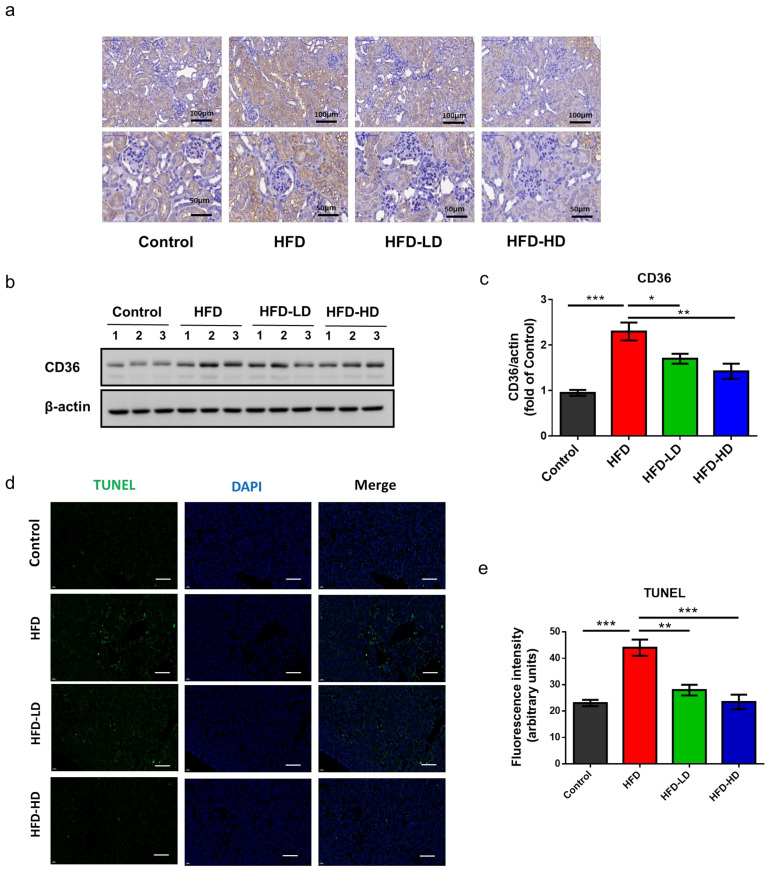
Empagliflozin attenuates CD36 expression in the kidney of HFD mice (*n* = 6). Empagliflozin attenuates CD36 expression in the HFD group, as shown by (**a**) immunohistochemical staining and (**b**,**c**) Western blotting. (**d**,**e**) Representative images and quantitative results of TUNEL staining show that empagliflozin attenuates cell apoptosis (green color) in a dose-dependent manner (scale bars = 50 μm). The histograms represent mean ± standard error (SE). Statistical analysis was performed with one-way ANOVA. * *p* < 0.05, ** *p* < 0.01, *** *p* < 0.001 vs. HFD in each group. HFD, high-fat diet; LD, low-dose empagliflozin; HD, high-dose empagliflozin.

Notably, CD36 is the major receptor for the uptake of oxidized low-density lipoprotein (OxLDL) and FFA. Accordingly, we examined the expression of CD36 in kidney tissues. Immunohistochemical staining revealed that HFD increased CD36 expression in the tubulointerstitial area, whereas empagliflozin decreased CD36 expression in the kidneys of HFD-fed mice (Figure 3a). In addition, western blotting for CD36 protein expression corroborated the immunohistochemical findings (Figure 3b,c). Next terminal deoxynucleotidyl transferase dUTP nick end labeling (TUNEL) stain was applied to examine cell apoptosis in the kidney. HFD increased TUNEL positive cells and empagliflozin ameliorated cell apoptosis in a dose-dependent manner (Figure 3d,e).

### 2.4. Empagliflozin Decreased Palmitic Acid-Induced CD36 Expression and Cell Death in a Proximal Tubular Cell Line (HK-2)

**Figure 4:**
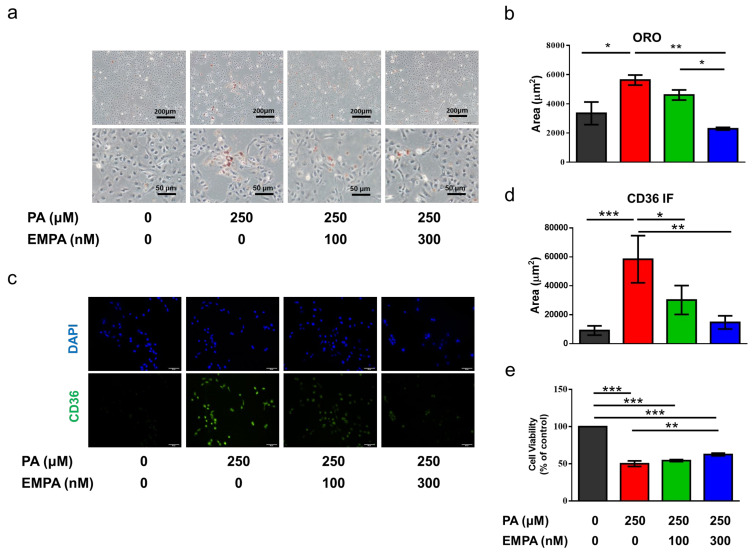
Empagliflozin ameliorates PA-induced lipid deposition, CD36 expression, and cell death in a proximal tubular cell line (HK-2). (**a**,**b**) Based on Oil red O-stained images, empagliflozin prevents lipid deposition in PA-treated HK-2 cells in a dose-dependent manner (scale bars at upper panel = 200 μm, lower pane l= 50 μm). (**c**) Immunofluorescent staining shows the upregulation of membranous CD36 (green) in the PA-treated group, while empagliflozin attenuates its expression. Scale bar = 50 μm (**d**) Quantitative results for immunofluorescent intensity are shown as mean ± standard error (SE) from 6 experiments. (**e**) Based on the cell viability test (*n* = 6), high-dose empagliflozin ameliorates PA-induced cell death. The histograms represent mean ± SE; Statistical analysis was performed with one-way ANOVA. * *p* < 0.05, ** *p* < 0.01, *** *p* < 0.001. Original magnification: 200×. EMPA, empagliflozin, PA, palmitic acid.

Previous studies using HFD animal models have demonstrated lipid accumulation in tubular areas [21] For comprehensively elucidating the molecular mechanism, we treated HK-2 cells (human renal proximal tubular cell line) with palmitic acid (PA), the major saturated FFA in obese mice to imitate an HFD-condition in vitro. Oil red O staining revealed that intracellular fatty acid accumulation was increased in PA-treated HK-2 cells, whereas empagliflozin attenuated this phenomenon (Figure 4a,b). Furthermore, treatment with PA enhanced CD36 expression; however, empagliflozin reduced CD36 expression in a dose-dependent manner (Figure 4c,d). On assessing cell viability, we observed that empagliflozin attenuated palmitate-induced cell death (Figure 4e).

### 2.5. Empagliflozin Abolished HFD- and PA-Induced Inflammation in Proximal Tubular Cells

**Figure 5:**
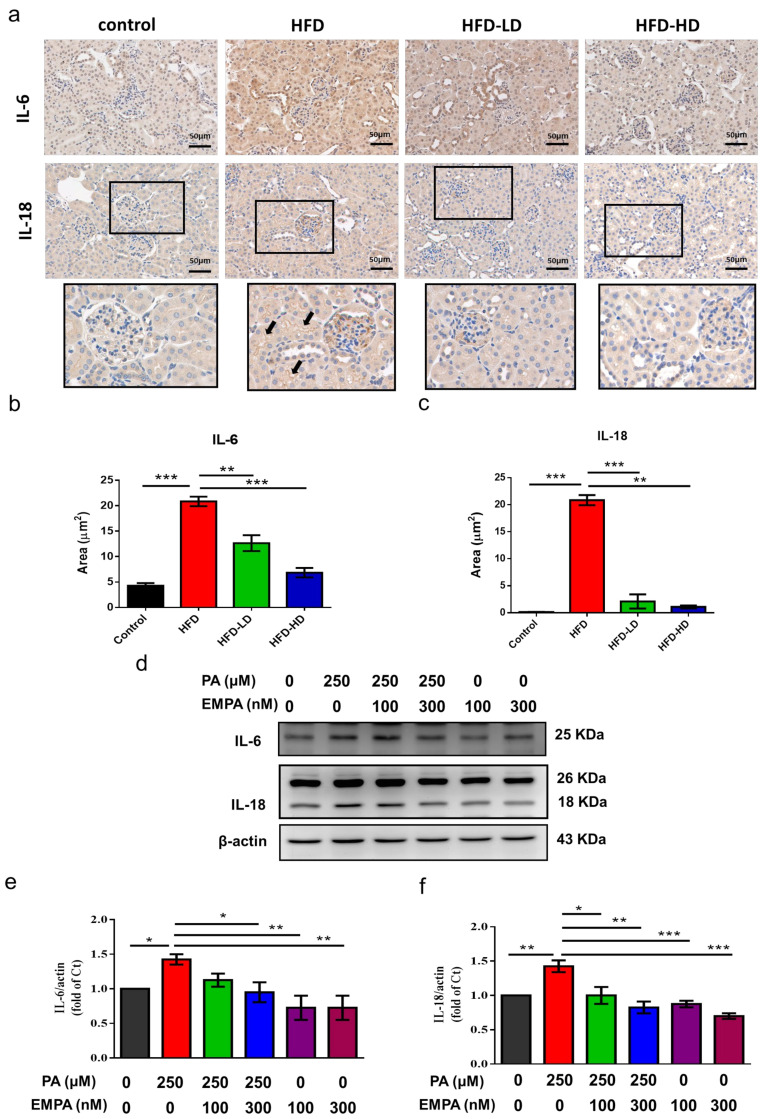
Empagliflozin abolishes HFD- and PA-induced cytokine production in the kidney section and proximal tubular cells. (**a**) HFD increased IL-6 expression in the tubular areas and IL-18 over lumens of tubules (black arrows) and glomerular areas. Both low and high dose of empagliflozin attenuated HFD-induced IL-6 and IL-18 expression. (**b**,**c**) Quantitative results for immunohistochemistry intensity are shown as mean ± standard error (SE) from six experiments. (**d**–**f**) HK-2 cells were cultured in medium with 1% bovine serum albumin and PA (250 μM), with or without low-dose (100 nM) or high-dose (300 nM) empagliflozin. High-dose empagliflozin attenuates PA-related upregulation of inflammatory cytokines IL-6 and IL-18. Statistical analysis was performed with one-way ANOVA. * *p* < 0.05, ** *p* < 0.01, *** *p* < 0.001. PA, palmitic acid; EMPA, empagliflozin.

We next evaluated HFD- and PA-induced inflammation. Accordingly, we examined the expression levels of inflammatory cytokines, interleukin (IL)-6 and IL-18. HFD increased IL-6 expression over renal tubular areas and IL-18 over glomerular and lumen of renal tubules. Both low and high dose of empagliflozin ameliorates HFD-induced IL-6 and IL-18 expression (Figure 5a–c).

Similarly, both low- and high-dose empagliflozin attenuated PA-induced upregulation of IL-6 and IL-18 in HK-2 cells (Figure 5). However, no further decrease of IL-6 and IL-18 in the absence of PA, which could be explained by low inflammatory status at baseline.

### 2.6. Empagliflozin Ameliorated Peroxisome Proliferator-Activated Receptor (PPAR)-γ Expression and Phosphorylation in HFD-Treated Kidneys and PA-Treated HK-2 Cells

**Figure 6:**
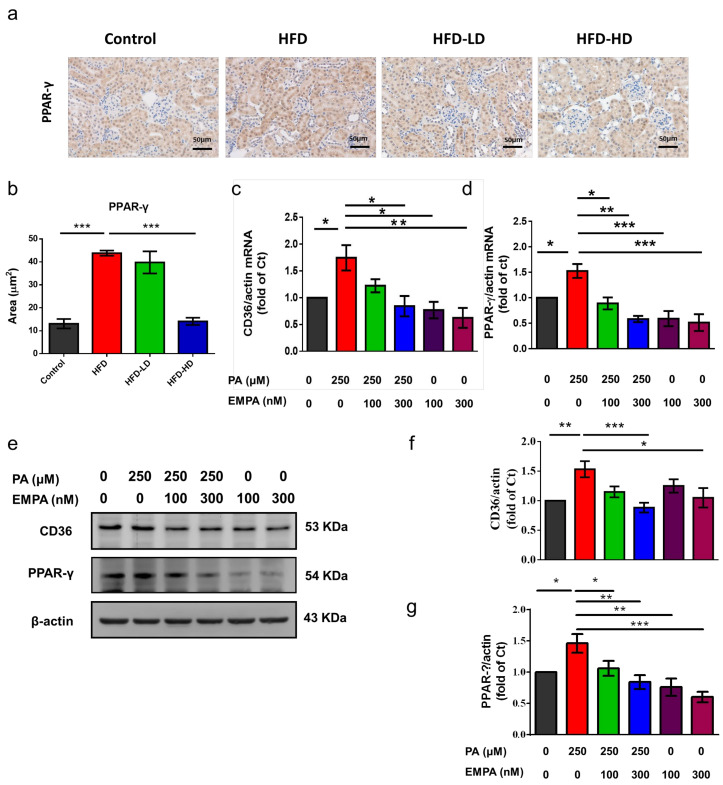
Empagliflozin treatment downregulates PPAR-γ expression in the kidney of HFD-fed mice and PA-treated HK-2 cells. (**a**,**b**) In the mouse kidney, high-dose empagliflozin attenuates PPAR-γ expression, as shown by immunohistochemical staining. (**c**) HK-2 cells were cultured in medium with 1% bovine serum albumin, PA (250 μM), and low-dose (100 nM) or high-dose (300 nM) empagliflozin. High-dose empagliflozin significantly reduces the expression levels of CD36 mRNA (**c**) and protein (**e**,**f**). Empagliflozin also reduces PPAR-γ mRNA (**d**) and protein expression (**e**,**g**). The histograms represent mean ± standard error (SE) from 6 experiments. Statistical analysis was performed with one-way ANOVA. * *p* < 0.05, ** *p* < 0.01, *** *p* < 0.001. EMPA, empagliflozin; PA, palmitic acid; PPAR, peroxisome proliferator-activated receptor.

HFD increased the expression of PPAR-γ in the renal tubular areas and empagliflozin attenuated its expression in a dose-dependent manner (Figure 6a,b). In HK-2 cells, empagliflozin significantly reduced the PA-induced upregulation of CD36 mRNA (Figure 6c) and protein levels (Figure 6e,f). The CD36 production involves complex transcriptional regulation and post-translational modification [6,23]. As PPAR-γ is one of the most important transcription factors of CD36 [24], we next evaluated the mRNA and protein expression of PPAR-γ and determined whether PPAR-γ is essential for empagliflozin-mediated CD36 regulation. PA significantly increased the mRNA and protein expression of PPAR-γ. Both high- and low-dose empagliflozin significantly reduced PPAR-γ levels in PA-treated HK-2 cells (Figure 6d,e,g).

**Figure 7:**
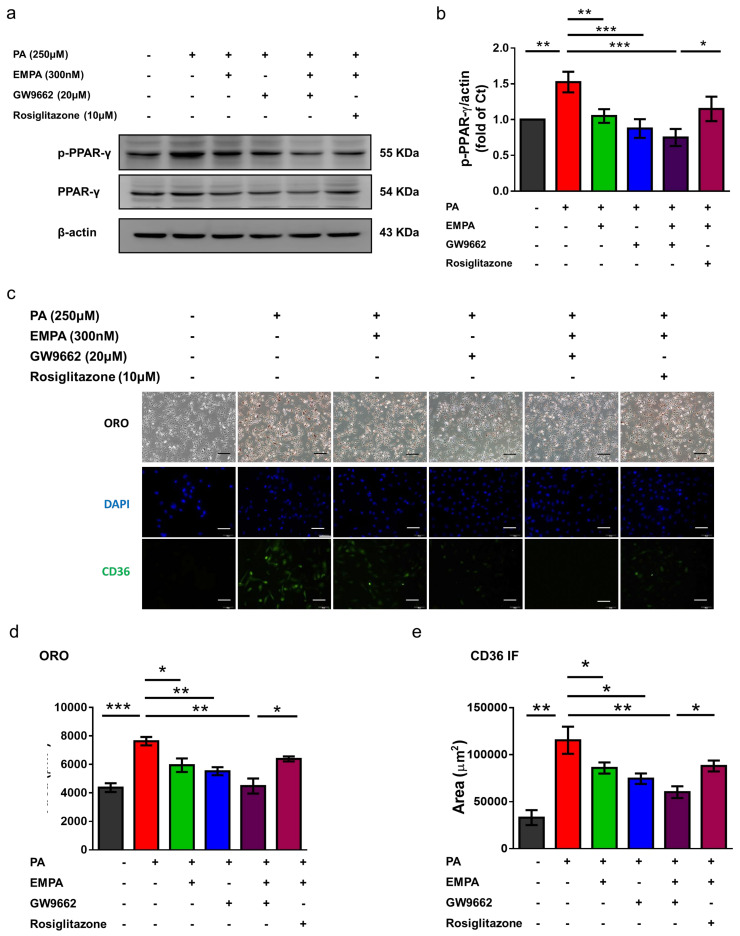
Empagliflozin mediates CD36 expression and reduces lipid accumulation via PPAR-γ phosphorylation in HK-2 cells. HK-2 cells were cultured in a medium with 1% bovine serum albumin, PA (250 μM), empagliflozin (300 nM), GW9662 (20 μM), and rosiglitazone (10 μM). (**a**,**b**) Western blotting reveals that empagliflozin and GW9662 attenuate PA-induced PPAR-γ phosphorylation, and rosiglitazone can reverse this effect. (**c**,**d**) Based on representative Oil red O-stained images, GW9662 and empagliflozin attenuate PA-induced lipid droplet deposition, and rosiglitazone can aggravate this parameter (Scale bars = 50 μm). (**c**,**e**) Based on the immunofluorescent assessment, GW9662 and empagliflozin attenuate PA-induced CD36 upregulation, and rosiglitazone can reverse this effect. The histograms represent mean ± standard error (SE) from 6 experiments. Statistical analysis was performed with one-way ANOVA. * *p* < 0.05, ** *p* < 0.01, *** *p* < 0.001. EMPA, empagliflozin; IF, immunofluorescence; ORO, Oil red O; PA, palmitic acid; PPAR, peroxisome proliferator-activated receptor.

To further determine whether empagliflozin directly inhibits CD36 via PPAR-γ, we used GW9662, a PPAR-γ inhibitor. Both GW9662 and empagliflozin reduced PPAR-γ phosphorylation (Figure 7a,b); however, cotreatment with GW9662 and empagliflozin did not further inhibit PPAR-γ phosphorylation. In contrast, rosiglitazone, a PPAR-γ agonist, reversed the inhibited phosphorylation (Figure 7a,b). In addition, Oil red O staining showed that both empagliflozin and GW9662 attenuated PA-induced intracellular lipid accumulation; this phenomenon was reversed by rosiglitazone (Figure 7c,d). Moreover, immunofluorescent images revealed that GW9662 decreased CD36 expression in HK-2 cells, whereas rosiglitazone increased this expression (Figure 7c,e). Collectively, these findings suggested that empagliflozin, at least partly, reduced CD36 expression via PPAR-γ.

## 3. Discussion

Obesity induces insulin resistance and adipocyte-specific metabolites, such as FFAs, which cause tubulointerstitial inflammation and injury. In the current study, our findings revealed that empagliflozin, an SGLT2 inhibitor, attenuated HFD-induced BW gain, as well as reduced lipid deposition and CD36 expression in the mouse kidney. Furthermore, empagliflozin downregulated PPAR-γ in vitro and decreased CD36 expression in PA-treated HK-2 cells. Consequently, empagliflozin attenuated PA-induced cell death and inflammation in renal proximal tubule cells. Thus, in addition to glucose control, empagliflozin may exert renal protection against lipotoxicity in proximal tubular cells by modulating the PPAR-γ/CD36 pathway.

Obesity-related metabolic syndrome involves low-grade inflammation, insulin resistance, hyperlipidemia and excess accumulation of harmful lipid metabolites in various organs and tissues, including the blood vessels, liver, and the kidney [25,26,27]. Our results revealed that empagliflozin significantly improved BW, insulin resistance, and lipid accumulation in the kidneys. SGLT2 inhibitors reportedly afford renoprotective effects owing to their glucose-lowering effects, possibly via a multifactorial mechanism. Systemically, Notably, SGLT2 inhibitors have been shown to lower blood pressure, decrease BW, and mitigate diabetic glomerular hyperfiltration [28,29,30]. Locally, SGLT2 inhibitors ameliorate glucotoxicity in renal tubular cells by suppressing glucose reabsorption [31] and reduce lipid nephrotoxicity, as shown in our results. Lipid nephrotoxicity indicates that hyperlipidemia, especially FFA or oxidized cholesterol, promotes the progression of chronic kidney disease [26,32]. Hosokawa et al. have shown that ipragliflozin, another SGLT2 inhibitor, can reduce ectopic lipid accumulation in tubular cells in non-alcoholic steatohepatitis (NASH)-model mice [33]. The selective SGLT2 inhibitor, JNJ 39933673, can also prevent renal lipid accumulation in db/db mice [21]. Similarly, Kahl et al. have demonstrated that empagliflozin can decrease hepatic steatosis in patients with type 2 DM [34]. The reduced lipid deposition and inflammation may be novel protective mechanisms underlying the pleiotropic effects of SGLT2 inhibitors in metabolic disorders.

CD36 is one of the major receptors mediating FFA uptake in proximal renal tubules [15]. CD36-dependent uptake of palmitate dose-dependently increases the levels of reactive oxygen species, inflammatory cytokine production, and cell death [35,36]. Herein, our results revealed that empagliflozin attenuated HFD- or PA-induced membranous CD36 expression and ameliorated PA-induced IL-6 and IL-18 expression and cell death in proximal tubular cells. Previously, we have demonstrated that blockade of CD36 by sulfosuccinimidyl oleate protects proximal tubular cells from palmitate-induced inflammasome activation and lytic cell death [35]. The pro-inflammatory cytokine IL-6 is associated with the transmigration of inflammatory cells into the interstitial space during diabetic tubulopathy [37]. IL-18, produced by the inflammasome, correlates with the degree of urinary albumin excretion and is overexpressed in the tubular epithelial areas in renal tissues during diabetic nephropathy [38,39]. Importantly, both IL-6 and IL-18 are upregulated by nuclear factor-κB (NF-κB) activation, triggering inflammation in cardiovascular and renal diseases [40,41]. Jaikumkao et. al. also demonstrated that dapagliflozin can also attenuated HFD-induced tumor necrotic factor (TNF)-α, IL-1β, iNOS and NF-κB activation, implicating that SGLT2 inhibition attenuates HFD-induced pro-inflammatory cytokines upregulation and M1 macrophage activation [42]. Empagliflozin might decrease CD36 expression and consequently mitigate downstream inflammatory cascades and cell death.

Our results demonstrated that empagliflozin suppressed CD36 mRNA expression in the presence of palmitate, which was further decreased in the absence of palmitate, suggesting that empagliflozin regulates CD36 expression at the transcriptional level. As the promoter of CD36 contains PPAR response elements (PPREs), CD36 transcription can be modulated by PPAR-α and PPAR-γ ligands [43,44]. In order to clarify the effect of empagliflozin on PPAR-γ, we employed a PPAR-γ antagonist (GW9662) and agonist (rosiglitazone). Like empagliflozin, GW9662 decreased CD36 expression, and rosiglitazone diminished empagliflozin-induced CD36 downregulation. PPAR-γ overexpression or activation by troglitazone reportedly induces CD36 expression and stimulates foam cell formation in vascular smooth muscle cells [45]. Cotreatment with GW9662 and empagliflozin failed to further downregulate CD36 expression when compared with empagliflozin alone, thus indicating that empagliflozin regulates CD36, at least partly, via PPAR-γ.

Certain limitations need to be acknowledged. Although the important roles of CD36 on metabolic disorder-induced proximal tubulopathy has been emphasized in many studies [13,15], FFA transports in renal proximal tubular cells also involve fatty acid transport -1 & -2, and plasma membrane fatty acid binding protein [15,46]. The beneficial effects of SGLT2 inhibition in lipotoxicity in renal proximal tubular cells can be partly explained by the regulation of CD36. Hence, it is mandatory to check other FFA transports in the future study. Moreover, we have only demonstrated the inhibition of CD36 by SGLT2 inhibitors. Despite not naturally seen, the beneficial effects of SGLT2 inhibitors on lipotoxicity would be more confirmed in a CD36 over-expressed cell model.

In conclusion, the current study illustrates that empagliflozin protects renal proximal tubular cells from HFD- or FFA-induced lipotoxicity. Empagliflozin downregulates CD36 via PPAR-γ and ameliorates palmitate-induced downstream inflammation and cell death. Thus, our study furnishes a novel mechanism through which empagliflozin confers pleiotropic renal protective effects.

## 4. Materials and Methods

### 4.1. Animal Model

Six-week-old male C57BL/6 mice were fed either a CD (10% of total calories from fat) or HFD with sucrose (58% of total calories from fat) (Research Diet, New Brunswick, NJ, USA) for 12 weeks. Mice were allocated into four groups: (1) CD, (2) HFD, (3) HFD plus low-dose empagliflozin (3 mg/kg/day), and (4) HFD plus high-dose empagliflozin (10 mg/kg/day) (Boehringer-Ingelheim, Ingelheim, Germany). Empagliflozin was administered by gavage for 12 weeks. At the end of the dietary period, mice were euthanized, and the kidneys were obtained for analyses [35]. All experimental procedures involving animals were approved by the Institutional Animal Case and Use Committee of the Kaohsiung Chang Gung Memorial Hospital (IACUC number: 2019060301, 2017032803).

### 4.2. Cell Cultures

HK-2 cells, human renal proximal tubular cells, were purchased from the American Type Culture Collection (Manassas, VA, USA) and maintained in RPMI 1640 medium supplemented with 10% (*v*/*v*) fetal bovine serum, 2 mM glutamine, 100 U/mL penicillin, and 100 μg/mL streptomycin. The experimental medium was prepared with a serum-free medium, and 10% fetal bovine serum was replaced with 1% (*w*/*v*) bovine serum albumin (BSA). PA (Cayman Chemical, Ann Arbor, MI, USA) was dissolved in dimethyl sulfoxide (DMSO) by heating at 60 °C to yield a stock concentration of 1 M. PA was diluted to 250 µM in RPMI medium and conjugated with 1% BSA for experimental treatments. Empagliflozin was diluted to 100 nM and 300 nM in RPMI medium for the experimental treatments.

### 4.3. Cell Viability

Cell viability was determined using the crystal violet assay. Briefly, HK-2 (5 × 10^3^) cells were seeded in each well of a 96-well cell plate and pretreated with empagliflozin (0, 100, and 300 µM) for 3 h, followed by PA (250 µM) for totally 24 h. After washing three times with phosphate-buffered saline, 100 μL of 4% formaldehyde was added and incubated for 30 min at 37 °C. Then, samples were washed with PBS three times, the suspension was removed, and 100 μL crystal violet (Sigma-Aldrich, St. Louis, MO, USA) was added to each well for 2 h on a shaker at room temperature. Distilled H_2_O was added to remove excess crystal violet, and the plate was then dried. Samples were re-dissolved in 100 µL DMSO on a shaker at room temperature and were measured using an ELISA reader (MQX200; BioTek, Winooski, VT, USA) at a wavelength of 570 nm.

### 4.4. Western Blotting

The harvested cells were lysed using RIPA buffer (Sigma-Aldrich) according to the manufacturer’s instructions. The protein concentrations were measured with Pierce™ BCA Protein Assay Kit #23225 (Thermo Fisher Scientific Inc., Waltham, MA, USA). Forty μg of protein were loaded for sodium dodecylsulfate-polyacrylamide gel electrophoresis and transferred to nitrocellulose membrane. After blocking with 5% skim milk in tris-buffered saline, the membrane was incubated with mouse anti-CD36 (1:2000; TA500921, OriGene, Rockville, MD, USA), rabbit anti-IL-18 (1:1000; ab191152, Abcam, Cambridge, MA, USA), mouse anti-IL-6 (1:1000; ab9324, Abcam), and mouse anti-PPAR-γ (1:1000; sc-7273, Santa Cruz Biotechnology Inc., Dallas, TX, USA) overnight at 4 °C. The membrane was washed with tris-buffered saline and incubated with their corresponding horseradhish peroxidase-conjugated secondary antibodies (1:5000) for 1 h at room temperature. An enhanced chemiluminescence system (GE Healthcare, Little Chalfont, UK) was used to detect target proteins. The protein expression was normalized with the signal obtained with β-actin expression [35].

### 4.5. Immunohistochemistry and Immunofluorescent Staining

Mouse kidneys were fixed with 4% paraformaldehyde and embedded in paraffin. Sections 5 mm thick were sliced and then rehydrated. The sections were incubated with 3% H_2_O_2_ for 10 min at room temperature, blocked with Ultra V block (Thermo Fisher Scientific Inc.) and incubated overnight at 4 °C with primary antibodies targeting CD36 (1:200; MA5-14112, Thermo Fisher Scientific Inc.), mouse anti-IL-6 (1:50; sc-57315, Santa Cruz Biotechnology Inc.), mouse anti-IL-18 (1:50; sc-7954, Santa Cruz Biotechnology Inc.) and mouse anti-PPAR-γ (1:50; sc-7273, Santa Cruz Biotechnology Inc.). The sections were then treated with mouse- and rabbit-specific HRP/DAB detection kits (TL-060-QHD; Thermo Fisher Scientific Inc.), counterstained with hematoxylin and observed under a microscope (Olympus, Tokyo, Japan).

The HK-2 cells were fixed with 4% formaldehyde for 15 min. After washed with PBS three times, samples were permeabilized with 0.1% Triton X-100 in PBS for 10 min at room temperature. To block unspecific binding of antibodies, cells were treated with 1% BSA in PBS for 1hr. The cells were then incubated with primary antibody at 1:50 dilution in PBS overnight at 4 °C. After washed with PBS three times, samples were incubated with fluorochrome-conjugated secondary antibody at 1:500 dilution in PBS for 1 h at 37 °C in dark. After washed with PBS three times, samples were incubated with 1 μg/mL DAPI. After washed with PBS three times, the samples were mounted with a drop of mounting medium. Fluorophore-conjugated secondary antibodies (Alexa Fluor 488 goat anti-mouse and 594 goat anti-rabbit, and Alexa Fluor 594 streptavidin; Thermo Fisher Scientific Inc.) were used to evaluate fluorescent signaling. Images were obtained using a fluorescence microscope (BX51; Olympus). The images of immunohistochemistry and immunofluorescent staining were analyzed by ImageJ (1.53 k, NIH, Bethesda, MD, USA)

### 4.6. Quantitative Reverse-Transcription Polymerase Chain Reaction

HK-2 cells were harvested and total RNA was extracted with the Total RNA Mini Kit (RB100) (Geneaid, New Taipei, Taiwan) according to the manufacturer’s protocol. RNA concentration was determined by NanoDrop-1000 spectrophotometer (Thermo Fisher Scientific Inc.). Changes in CD36 and PPAR-γ mRNA levels were determined by quantitative real-time polymerase chain reaction (PCR) using Roche LightCycler 480^®^ (Roche Applied Science, Mannheim, Germany) apparatus and LightCycler 480 SYBR Green I Master kit (Roche Applied Science), with 0.5 µM each primer for CD36 (forward primer: 5-CTCTTTCCTGCAGCCCAATG-3; reverse primer: 5-CTGCCACAGCCAGATTGAGA-3; Invitrogen, USA) and PPAR-γ (forward primer: 5-TGCACTGGAATTAGATGACAGC-3; reverse primer: 5′-TCCGTGACA ATCTGTCTGAGG-3′; Invitrogen, Carlsbad, CA, USA). The expression of β-actin was used as the internal control data normalization [47].

### 4.7. Oil Red O Staining

Neutral lipids in HK-2 cells and frozen mouse kidney sections (5 mm in thickness) were accessed by Oil red O staining. The samples were fixed with 4% paraformaldehyde at 4 °C for 15–30 min. After fixative removed, the samples were rinsed with PBS and then allowed to air dry. In the meantime, oil red o stock was mixed at 3:2 ratio with ddH2O and then stood for 10 min. After filtered with fast filter, Oil red O was added with a 0.2 micron syringe filter to samples. After 15 min, the Oil red O was removed and the samples were rinsed with ddH20. The image was accessed by using an Olympus Bx4I microscope (original magnification, 40×) with an AxioCamMR5 photographic attachment (Zeiss, Gottingen, Germany) [48].

### 4.8. Terminal Deoxynucleotide Transferase-Mediated dUTP Nick End Labeling Assay

Frozen mouse kidney sections (5 mm in thickness) were fixed with 4% formaldehyde in PBS for 10 min. Apoptotic cells and DNA fragmentation were detected by the DeadEnd Fluorometric TUNEL System (Promega, Madison, WI, USA) according to manufacturer’s instructions. Images were obtained using a fluorescence microscope (BX51, Olympus) [35].

### 4.9. Measurement of Metabolic Parameters

(1) Fasting blood glucose levels were determined using a glucometer (Accu-Chek^®^ Active, Roche, Mannheim, Germany) in a blood sample collected from the tail vein after 6 h of fasting.
(2) IPGTT was performed at week 12 after fasting for 16 h. Glucose (2 g/kg BW) was intraperitoneally administered, and blood was collected from the tail tip of mice. Glucose concentration was measured using a glucometer, as described previously, at time point 0 (prior to glucose administration). In addition, blood glucose levels were assessed at 5, 15, 30, 60, 90, and 120 min after intraperitoneal injection.
(3) For performing the IPITT, food was withdrawn for 4 h, and then mice were intraperitoneally administered insulin (0.75 U/kg) at time 0. Blood sugar was assessed at 15, 30, 60, 90, and 120 min after intraperitoneal injection. [47]

### 4.10. Statistical Analysis

Data are presented as the mean ± standard error (SE). Differences between the two groups were identified using an unpaired t-test. Multiple comparisons were made using one-way analysis of variance (ANOVA) followed by Bonferroni’s test. For all tests, *p*-values ˂ 0.05 were considered statistically significant. All statistical analyses were carried out using GraphPad Prism version 6.00 (Graph Pad Software, Inc., San Diego, CA, USA).

## Data Availability

Not applicable.
